# Estimating incidence of type 1 and type 2 diabetes using prevalence data: the SEARCH for Diabetes in Youth study

**DOI:** 10.1186/s12874-023-01862-3

**Published:** 2023-02-14

**Authors:** Annika Hoyer, Ralph Brinks, Thaddäus Tönnies, Sharon H. Saydah, Ralph B. D’Agostino, Jasmin Divers, Scott Isom, Dana Dabelea, Jean M. Lawrence, Elizabeth J. Mayer-Davis, Catherine Pihoker, Lawrence Dolan, Giuseppina Imperatore

**Affiliations:** 1 Medical School OWL, Biostatistics and Medical Biometry, Bielefeld University, Universitätsstr. 25, Bielefeld, 33615 Germany; 2 Chair for Medical Biometry and Epidemiology, Faculty of Health/School of Medicine, Witten/Herdecke University, Witten, Germany; 3 Institute for Biometrics and Epidemiology, German Diabetes Center, Leibniz Center for Diabetes Research at the Heinrich Heine University, Düsseldorf, Germany; 4 Division of Viral Diseases, National Center for Infectious Respiratory Diseases, Centers for Disease Control and Prevention, Atlanta, USA; 5 Department of Biostatistics and Data Science, Division of Public Health Sciences, Wake Forest School of Medicine, Winston-Salem, North Carolina USA; 6 Division of Health Services Research, Department of Foundations of Medicine, New York University Langone School of Medicine, Mineola, NY USA; 7 Lifecourse Epidemiology of Adiposity and Diabetes (LEAD) Center, Department of Epidemiology, Colorado School of Public Health, University of Colorado, Denver, CO USA; 8 Division of Diabetes, Endocrinology & Metabolic Diseases, National Institute of Diabetes and Digestive and Kidney Diseases, National Institutes of Health, Bethesda, MD USA; 9 Departments of Nutrition and Medicine, Gillings School of Global Public Health and School of Medicine, University of North Carolina, Chapel Hill, NC USA; 10 Department of Pediatrics, University of Washington, Seattle, WA USA; 11 Division of Endocrinology, Department of Pediatrics, Cincinnati Children’s Hospital, University of Cincinnati College of Medicine, Cincinnati, OH USA; 12 Division of Diabetes Translation, Centers for Disease Control and Prevention (CDC), National Center for Chronic Disease Prevention and Health Promotion, Atlanta, USA

**Keywords:** Adolescents, Children, Epidemiology, Illness-death-model, Surveillance, Type 1 diabetes, Type 2 diabetes

## Abstract

**Background:**

Incidence is one of the most important epidemiologic indices in surveillance. However, determining incidence is complex and requires time-consuming cohort studies or registries with date of diagnosis. Estimating incidence from prevalence using mathematical relationships may facilitate surveillance efforts. The aim of this study was to examine whether a partial differential equation (PDE) can be used to estimate diabetes incidence from prevalence in youth.

**Methods:**

We used age-, sex-, and race/ethnicity-specific estimates of prevalence in 2001 and 2009 as reported in the SEARCH for Diabetes in Youth study. Using these data, a PDE was applied to estimate the average incidence rates of type 1 and type 2 diabetes for the period between 2001 and 2009. Estimates were compared to annual incidence rates observed in SEARCH. Precision of the estimates was evaluated using 95% bootstrap confidence intervals.

**Results:**

Despite the long period between prevalence measures, the estimated average incidence rates mirror the average of the observed annual incidence rates. Absolute values of the age-standardized sex- and type-specific mean relative errors are below 8%.

**Conclusions:**

Incidence of diabetes can be accurately estimated from prevalence. Since only cross-sectional prevalence data is required, employing this methodology in future studies may result in considerable cost savings.

**Supplementary Information:**

The online version contains supplementary material available at 10.1186/s12874-023-01862-3.

## Background

In epidemiologic practice, assessing serial incidence rates is typically more difficult than calculating the prevalence of a disease [1]. For this reason, statistical methods aiming to use prevalence data to estimate incidence are of special interest for disease surveillance. Such approaches may be alternatives to developing disease registers for chronic conditions such as diabetes mellitus, which are time-consuming and costly. Especially with regard to quantifying the burden of chronic diseases in youth, type 1 and type 2 diabetes mellitus have been the focus of extensive surveillance efforts and are therefore a good condition to evaluate statistical methods for estimating incidence rates from prevalence data.

Type 2 diabetes, resulting from insulin resistance (most commonly associated with obesity) and inadequate beta cell compensation, is the most common form of diabetes in adults. Type 1 diabetes, resulting from an autoimmune attack on the insulin producing cells of the pancreas, is the most common form of youth-onset diabetes and one of the most common chronic diseases of childhood. Type 1 diabetes affects about 167,000 young people in the U.S. resulting in a prevalence of 1.93% in 2009 [2]. With the rise in obesity in youth over the past three decades, type 2 diabetes in youth has also become a pediatric health concern [3–5], although still less common than type 1 diabetes in this group. Due to a comparatively small absolute prevalence, observing new cases and estimating incidence rates of type 1 diabetes among adults, and type 2 diabetes among youth, can be challenging.

Since the incidence and prevalence of type 1 and type 2 diabetes are increasing in the U.S. population aged < 20 years [6, 7], surveillance at population level has become an important tool for identifying potential risk factors, evaluating the efficacy of prevention programs, and planning for future health care needs and expenditures. However, surveillance efforts are costly and time consuming [8]. Measurement of incidence is one of the goals of surveillance of non-diseased populations and usually requires a time frame in which a population at risk is observed and followed up to develop the disease. Prevalence studies are easier to design because follow up examinations are not necessary, and they are less expensive than studies to estimate incidence rates [1]. Thus, an accurate method of estimating incidence rates from prevalence data is likely to improve efficiency of public health surveillance efforts.

The aim of these analyses was to determine whether prevalence can be used to estimate average annual incidence rates using data from the SEARCH for Diabetes in Youth study [9]. To this end, we employed SEARCH prevalence data to estimate average incidence rates between 2001 and 2009 based on a model developed by Brinks and Landwehr [10]. The estimated incidence rates were then compared to the observed SEARCH annual age-, sex-, race/ethnicity-, and type-specific incidence data.

## Methods

### Data sources

This study is based on data from the SEARCH for Diabetes in Youth study, which was a multicenter study from 2001 to 2020, conducting population-based ascertainment of clinically diagnosed, non-gestational diabetes cases among youth aged less than 20 years in the United States. In short, five centers across the US report prevalent and incident diabetes cases which were identified by participating endocrinologists (pediatric and adult), as well as other health care providers, hospitals, health systems, community health centers, clinical and administrative data systems and electronic medical records. Validation of diagnosis of diabetes was done by review of a physician’s diagnosis of diabetes in the medical records. For the population under surveillance, case ascertainment was estimated > 90% complete [7]. Diabetes cases were ascertained from geographically defined populations in Ohio, Colorado, South Carolina and Washington, Indian Health Service beneficiaries from selected American Indian populations, and enrollees in a managed health care system in California. Institutional review board(s) for each site approved the study protocol. For a more detailed description of the study we refer to Hamman et al. and The SEARCH Study Group [9, 11].

### Statistical analysis

#### Prevalence

Prevalence of type 1 and type 2 diabetes was available separately for 2001 and 2009. Assessment methods have been described elsewhere [6]. To determine prevalence, the numerator included all prevalent diabetes cases in 2001 or 2009 who were < 20 years of age on December 31, 2001 or 2009, resident of the SEARCH geographic sites, Indian Health Service beneficiaries, or enrollees in the participating health system. Institutionalized individuals and active duty military were not eligible. Using self-reports or medical records, race/ethnicity was available in 2001 and 2009 for 94.9% and 97.3% of the study participants, respectively. Missing data on race/ethnicity were imputed via geocoding (5.1% in 2001 and 2.7% in 2009).

Denominators were based on the number of participants aged < 20 years who were residents of the geographic study areas, Indian Health Service beneficiaries, or members of the participating health system in 2001 or 2009.

For the present analysis, numerator and denominator were grouped into four racial/ethnic groups: Hispanic, non-Hispanic white (NHW), non-Hispanic black (NHB), and non-Hispanic other (other).

Medical records were used to determine date of diagnosis, diabetes type and demographic information. Diabetes type-specific prevalence in 2001 and 2009 were estimated for each age from 0 to 19 years, sex, and race/ethnicity.

#### Incidence

Incident cases of type 1 and type 2 diabetes were ascertained from 2002 onwards in SEARCH. Between 2002 and 2008, 6,995 incident cases of type 1 diabetes and 1,655 incident type 2 diabetes cases were identified.

Assessment of diabetes incidence in SEARCH has been described elsewhere [7]. In short, all incident diabetes cases aged less than 20 years on December 31 of the year an incident case entered SEARCH (index year) were included. Self-report (81%), medical records (16%), and geocoding (3%) were used to assess race/ethnicity. Individuals who were aged less than 20 years on December 31 of the index year and were civilian residents of the geographic study areas, Indian Health Service beneficiaries of participating American Indian tribes, or members of the participating health plan were part of the annual denominators for determining incidence.

Pooling the data across the five centers, annual incidence rates were given per 100,000 person years. Diabetes type-specific incidence rates were estimated for each age ranging from 0 to 19 years, sex, race/ethnicity, and calendar year.

#### Mathematical model

**Fig. 1:**
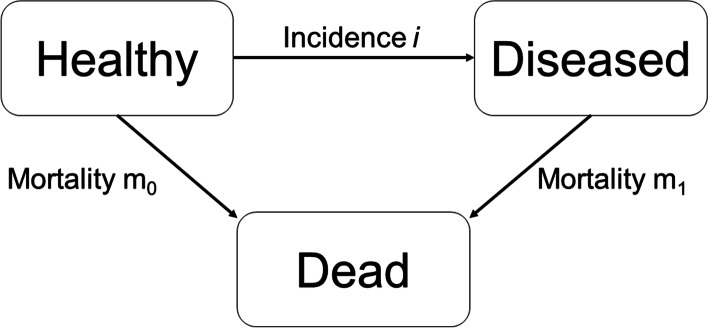
Illness-death model

Figure 1 depicts the illness-death model which was used to estimate the incidence rates of type 1 and type 2 diabetes in youth between 2001 and 2009 based on prevalence data in 2001 and 2009. The model consists of three states: Healthy (with respect to the considered disease), Diseased, and Dead. The transition rates between the states are the incidence $i$ of the disease, and the mortality rates ${m}_{1}$ and ${m}_{0}$ with and without the disease, respectively. All transition rates depend on age ($a$) and calendar time ($t$). As shown by Brinks and Landwehr [12] the model is governed by a system of partial differential equations (PDE) that relates the temporal change of age-specific prevalence to age-specific incidence and mortality rates. Using the general mortality $m=m\left(t, a\right)=\left(1-p\right){\times m}_{0}(t,a)+p\times {m}_{1}(t,a)$ of the population where $p=p\left(t,a\right)$ denotes the prevalence, and the mortality rate ratio $R=R\left(t,a\right)={m}_{1}\left(t,a\right)/{m}_{0}\left(t,a\right)$, the PDE is given by

1 $$\left(\partial /\partial t+\partial /\partial a\right)p=\left(1-p\right)\times \left[i-m\times p\times \left(R-1\right)/(1+p\times \left(R-1\right))\right]$$

For the present analysis, $a$ represents all ages from 0 to < 20 years and $t$ covers all years from 2001 to 2009. As mortality rates of youth in the chosen age range are very low in individuals with diabetes as well as without [13], the difference between these rates is low and therefore negligible. Consequently, we assumed that both mortality rates are equal, resulting in a difference ${m}_{1}-{m}_{0}$ = 0. It has been shown that this assumption does not affect the final estimates [14]. Finally, the PDE simplifies to

2 $$\left(\partial /\partial t+\partial /\partial a\right)p=\left(1-p\right)\times i$$

Observed prevalence estimates in 2001 were used as starting values for solving the PDE (2). Furthermore, prevalence from 2009 was used as input value aiming to estimate the average incidence rates between 2001 and 2009. To evaluate the accuracy of the estimated incidence, 95% bootstrap confidence intervals were calculated. Doing so, we sampled initial as well as input values from the distribution of the age-specific prevalence in 2001 and 2009, respectively. Based on the sampled values, the average annual incidence rates between 2001 and 2009 were estimated using Eq. (2). This procedure was repeated 5,000 times. Finally, we used the 2.5 and 97.5 percentile of the resulting distribution as the lower and upper limit of the 95% confidence interval of the type-, age-, race/ethnicity- and sex-specific average annual incidence rates between 2001 and 2009. For comparison of the estimated average incidence rates (E) with the observed average incidence rates (O), we present relative errors in percent, i.e. 100% × (E-O)/O, together with the associated 95% bootstrap confidence intervals. For easier comparison, incidence rates were additionally standardized by age with standardization weights from US general population in the year 2000. All analyses were done using the software R (The R Foundation of Statistical Computing).

## Results

### Model input: prevalence in 2001 and 2009

**Fig. 2:**
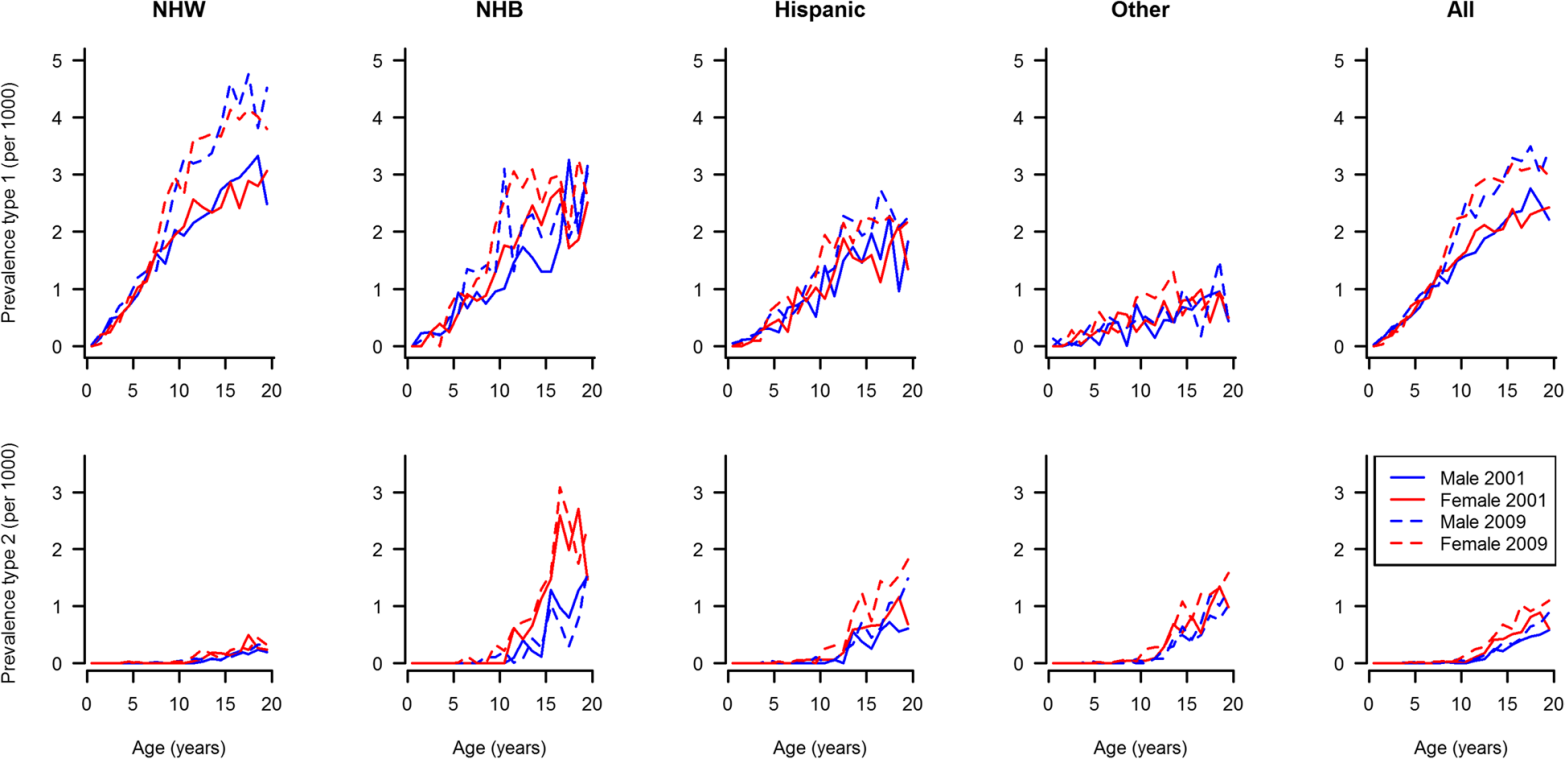
Prevalence of type 1 and type 2 diabetes in U.S. youth aged 0–19 years by age, sex, and race/ethnicity in 2001 and 2009. Abbreviations: NHW, non-Hispanic white; NHB, non-Hispanic black

Figure 2 depicts the observed prevalence of type 1 and type 2 diabetes by age, sex, and race/ethnicity in the years 2001 and 2009. From 3,345,777 and 3,458,969 youth aged < 20 years, 4,832 and 6,626 cases of type 1 diabetes were identified in 2001 and 2009, respectively. For both years and sexes, prevalence of type 1 and type 2 diabetes increased with age. For type 1 diabetes, the prevalence was higher in NHW and NHB compared to Hispanics and other race/ethnicity groups. With respect to type 2 diabetes, 586 and 1,140 cases were identified in 2001 and 2009, respectively. Prevalence was lowest in the ages below 10 years, increasing afterwards. The highest prevalence was observed among NHB females.

### Comparison of model-estimated with observed annual incidence rates between 2001 and 2009

**Fig. 3:**
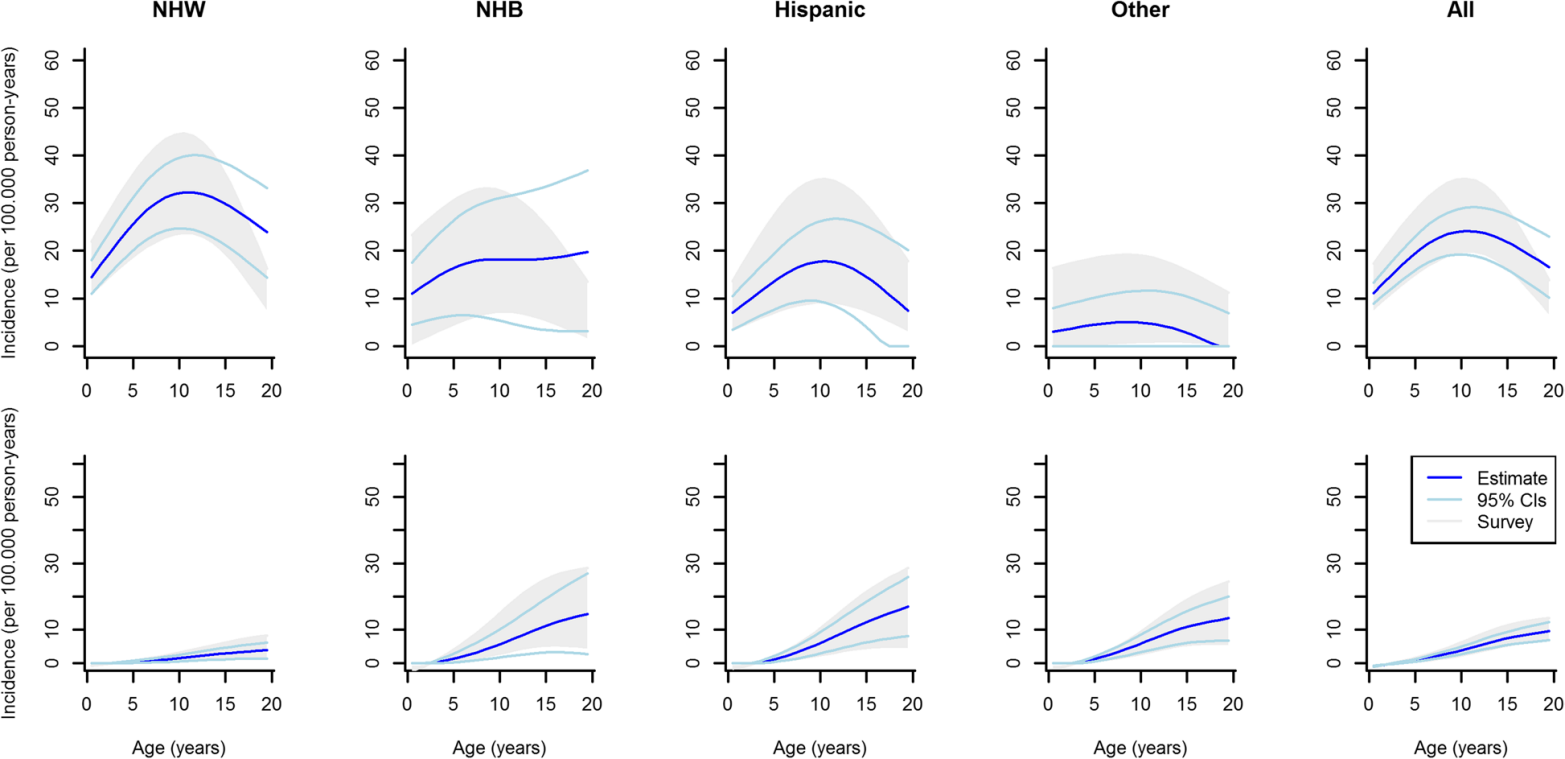
Observed minimum and maximum (grayish area) and estimated (solid line) incidence of type 1 diabetes (upper row) and type 2 diabetes (bottom row) in U.S. males between 2001 and 2009 by age and race/ethnicity, light blue lines represent 95% confidence intervals. Abbreviations: NHW, non-Hispanic white; NHB, non-Hispanic black

Between 2001 and 2009, a total of 9,118 new diabetes cases within 74,413,003 person years were reported by SEARCH for the youth population aged < 20 years. Maximum and minimum observed as well as model-estimated incidences between 2001 and 2009 of type 1 and type 2 diabetes by age, and race/ethnicity are depicted in Figs. 3 (males) and 4 (females). Note that SEARCH reports incidence annually. However, our aim was to estimate incidence for the 2001–2009 time period. Therefore, our estimate can be seen as an average rate over the annually reported incidences. Thus, we show the model estimates as well as the minimum and maximum observed incidence from SEARCH. With respect to type 1 diabetes, incidence increased for all race/ethnicity groups up to an age of approximately 10 years. Beyond age 10 years, a decrease is observed. For type 2 diabetes, incidence increased over the whole age range whereas lowest rates are observed for NHW youth. Wider confidence intervals are observed for higher age groups which is due to a lower number of new cases and a decreasing incidence. Table 1 shows the observed and estimated average age-standardized incidence rates, as well as the relative error, stratified by sex and diabetes type. Absolute values of the mean relative errors are below 8%. Overall, the mathematical model estimates the mean incidence rate between 2001 and 2009 very well. In the online additional information, we show the numerical results for type 1 and type 2 diabetes stratified by sex with respect to the observed and estimated average incidence rates as well as their relative errors with corresponding 95% confidence intervals (see Tables S1 and S2). The findings suggest that for type 1 diabetes, the differences between observed and modelled incidence rates are in good agreement with respect to the relative errors for ages between 2 and 18. For the lowest (< 2 years) and highest (19–20 years) ages, we observed larger deviations between the averaged observed and estimated incidence. For type 2 diabetes differences between incidences are larger, especially for younger age groups where only a few cases were observed.

**Table 1:** Observed and estimated average age-standardized incidence rates with 95% confidence intervals. Standardization was done according to US general population in the year 2000

	Average observed incidence rate (per 100.000 person-years)		Average estimated incidence rate (per 100.000 person-years)		Relative error (in %)	
	Male	Female	Male	Female	Male	Female
Type 1 Diabetes	21.04 [21.00; 21.09]	20.26 [20.22; 20.31]	20.01 [18.47; 21.57]	18.67 [17.17; 20.21]	-4.88 [-12.28; 2.57]	-7.86 [-15.29; -0.20]
Type 2 Diabetes	4.09 [3.89; 4.29]	6.19 [6.09; 6.29]	4.13 [3.51; 4.74]	5.85 [5.13; 6.55]	0.81 [-14.51; 16.70]	-5.50 [-17.06; 6.31]

**Fig. 4:**
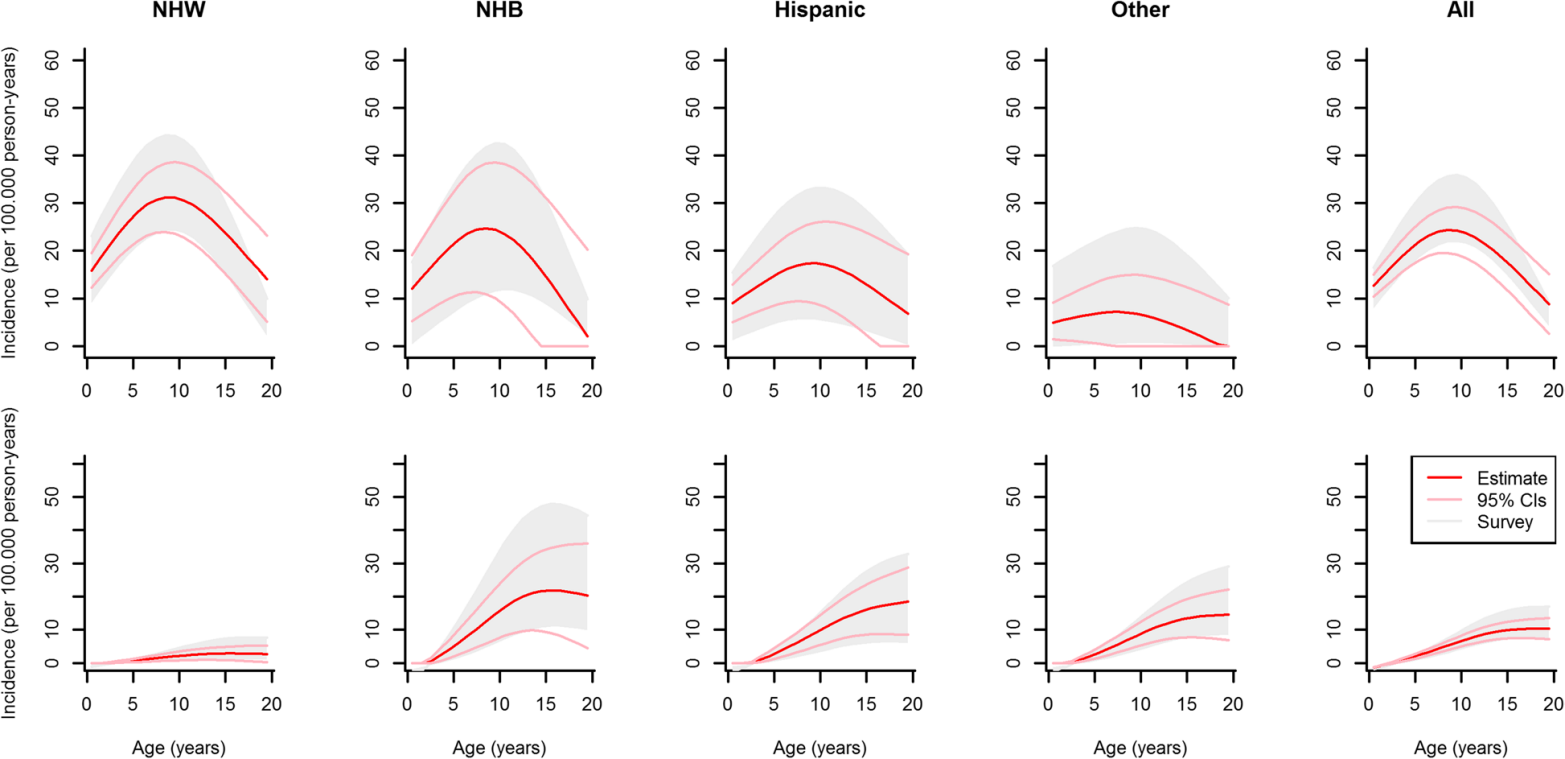
Observed minimum and maximum (grayish area) and estimated (solid line) incidence of type 1 diabetes (upper row) and type 2 diabetes (bottom row) in U.S. females between 2001 and 2009 by age and race/ethnicity, light pink lines represent 95% confidence intervals. Abbreviations: NHW, non-Hispanic white; NHB, non-Hispanic black

## Discussion

This study demonstrated that a PDE previously used to describe the illness-death model can be employed to estimate average annual incidence rates from prevalence data. Specifically, the estimated incidence between 2001 and 2009 obtained using a PDE approximates the average of the annually observed (true) incidence rates. The data suggest that employing the PDE is a valid approach for estimating incidence, potentially reducing cost and effort expended in surveillance of childhood diabetes.

The PDE is applicable to all chronic diseases and, moreover, can also be used in case remission rates and differential mortality rates are observed [15–18] It can also be applied in various ways. For example, we showed that excess mortality can be estimated based on prevalence and incidence while assessing the impact of the diagnostic accuracy of the underlying data set [19]. A validation study [20] using real world data as well as simulation studies [21] has proven the validity of the PDE and demonstrated that the results mainly depend on accuracy and validity of the input data. Overall, the method described here is superior over other methods with respect to bias (mean absolute error) [22]. To quantify precision of estimates, we used 95% bootstrap confidence intervals. Confidence intervals were smaller for type 2 diabetes and for larger race/ethnicity groups such as NHW. Higher uncertainty was observed for smaller subgroups with rare events. Alternatively, pooling data, for example across age groups, would improve precision of the estimates. Highest precision is indicated when pooling all strata together.

### Limitations

Regarding limitations of our study, we first assume that the very low observed mortality rates of diabetes among U.S. youth [13, 23] do not affect our estimates and can therefore be neglected, e.g. we assumed a relative risk of mortality of one. However, if mortality differs substantially between the population of diseased and non-diseased, it has to be incorporated into the model [17].

Second, eight years between the observed prevalence in 2001 and 2009 are used for estimating incidence. Hence, the PDE approach provides an average of the yearly incidences over this eight-year period. However, we do not have a direct estimate for this eight-year-average incidence rate because SEARCH has only yearly incidence data. Thus, strictly speaking, there is no ground truth to compare our estimate with. Nevertheless, using annually or bi-annually reported prevalence data will lead to incidence estimates for shorter time periods and to the possibility of assessing time trends.

Third, the PDE presented here assumes that the prevalence of type 1 and type 2 diabetes in migrants is similar to the resident population. Brinks and Landwehr published a model that can be used in case this assumption is heavily violated [10]. However, the annual number of immigrant youth in the U.S. is very low [24] which justifies our assumption.

Fourth, it would be favorable to include an additional state of undiagnosed diabetes, i.e. individuals with the disease that have not yet been identified, which is currently not done. For both type 1 and type 2 diabetes in youth the classification of undiagnosed diabetes appears to be a minor issue. Type 1 diabetes is associated with a rapid deterioration in glucose control and clear clinical symptoms. In contrast, in adults with type 2 diabetes there is a prolonged phase of asymptomatic diabetes prior to diagnosis. However, in population-based screenings for diabetes in youth, very few cases of undiagnosed (asymptomatic) diabetes were found [25, 26]. Adding other states to the model, like the different stages of type 1 diabetes, would be possible, but would significantly increase the complexity of the underlying mathematical methods and data requirements. This is therefore beyond the scope of the study.

### Future directions

Estimating incidence rates from prevalence data may be time-saving and potentially improves diabetes surveillance efforts. To this end, we suggest a possible way to complement estimates from costly cohort studies or registries to identify new cases. Instead, two cross-sectional studies that measure (point) prevalence are sufficient to obtain information about incidence. Accurately assessing prevalence and especially incidence rates is of high importance for establishing public health surveillance programs and to inform epidemiological as well as clinical research [27]. These numbers can be seen as the basis for estimating health service needs and related costs. Furthermore, it is expected that the number of observed persons on which prevalence is calculated, affects the estimation of the incidence. Therefore, the development of sample size calculation formulas would be the next step in this line of research.

## Conclusions

In summary, we demonstrated how a PDE that describes the illness-death model can be used to estimate average age-, sex-and race/ethnicity-specific incidence rates for youth-onset diagnosed type 1 and type 2 diabetes from prevalence data. This model represents an efficient way to improve childhood diabetes surveillance as it is time-saving compared to cohort studies or registries which are also often associated with higher costs when they are conducted. Furthermore, if prevalence is available for several points in time, it would be possible to estimate incidence rates between these points and to quantify trends. Such trends can be a basis for projecting future numbers of diabetes cases [28, 29] or for evaluating primary prevention programs for type 2 diabetes.

## Supplementary Information

**Additional file 1: S1 Table.** Observed and estimated average incidence rates of type 1 diabetes. **S2 Table.** Observed and estimated average incidence rates of type 2 diabetes.

## Data Availability

The data underlying this article cannot be shared publicly due to privacy of individuals that participated in the study. The data will be shared on reasonable request to the corresponding author. We additionally provide an artificial data set that mirrors the original data and can be used to reproduce the presented analysis and results. The full R-code and data set are available under https://doi.org/10.5281/zenodo.7543640.

## Abbreviations

NHB: Non-Hispanic black
NHW: Non-Hispanic white
PDE: Partial differential equation
