# Supplementary material for: Estimating incidence of type 1 and type 2 diabetes using prevalence data: the SEARCH for Diabetes in Youth study
Source: BMC Med Res Methodol. 2023 Feb 14;23:39. doi: 10.1186/s12874-023-01862-3 (PMC9930314; doi:10.1186/s12874-023-01862-3)
Supplement: Supplementary file 1 — Additional file 1: S1 Table. Observed and estimated average incidence rates of type 1 diabetes. S2 Table. Observed and estimated average incidence rates of type 2 diabetes. [file 12874_2023_1862_MOESM1_ESM.pdf]

Additional information to "Estimating incidence of type 1 and type 2 diabetes using prevalence data: the SEARCH for Diabetes in Youth Study"

S1 Table. Observed and estimated average incidence rates of type 1 diabetes

Age	Average observed incidence rate (per 100.000 person-years)		Average estimated incidence rate (per 100.000 person-years)		Relative error (in %)	
	Male	Female	Male	Female	Male	Female
0 - 1	2.05 [1.28; 3.30]	2.39 [1.53; 3.75]	11.1 [8.92; 13.3]	12.6 [10.3; 15.0]	304.66 [174.62; 477.89]	263.69 [158.60; 393.96]
1 - 2	19.4 [16.6; 22.6]	13.2 [10.9; 15.9]	13.1 [10.6; 15.6]	14.7 [12.1; 17.4]	-43.49 [-56.28; 30.74]	-22.58 [-40.75; -3.87]
2 - 3	15.5 [13.0; 18.4]	15.8 [13.3; 18.9]	15.0 [12.2; 17.8]	16.7 [13.8; 19.7]	-17.78 [-35.78; -0.54]	-17.80 [-35.20; -0.80]
3 - 4	18.2 [15.5; 21.3]	19.9 [17.0; 23.2]	16.9 [13.8; 20.0]	18.7 [15.4; 22.0]	-28.26 [-43.14; -12.94]	-21.65 [-36.85; -6.06]
4 - 5	19.4 [16.6; 22.6]	25.1 [21.8; 28.8]	18.6 [15.2; 22.0]	20.5 [16.9; 24.1]	-1.71 [-18.81; 15.28]	-12.22 [-26.34; 1.84]
5 - 6	21.6 [18.7; 25.0]	27.0 [23.6; 30.8]	20.2 [16.5; 23.9]	22.0 [18.1; 25.9]	7.06 [-11.24; 24.95]	-2.22 [-17.49; 13.01]
6 - 7	25.1 [21.9; 28.7]	24.9 [21.7; 28.6]	21.6 [17.6; 25.6]	23.2 [19.0; 27.4]	7.85 [-10.02; 25.13]	4.68 [-12.44; 21.74]
7 - 8	22.5 [19.5; 26.0]	27.7 [24.3; 31.6]	22.7 [18.4; 26.9]	24.0 [19.5; 28.5]	4.01 [-15.87; 22.97]	6.98 [-10.51; 24.22]
8 - 9	30.9 [27.4; 34.9]	29.6 [26.1; 33.7]	23.5 [19.0; 28.0]	24.3 [19.6; 29.1]	-14.44 [-29.64; 0.52]	0.71 [-15.69; 17.69]
9 - 10	28.2 [24.8; 32.0]	31.4 [27.8; 35.5]	24.0 [19.2; 28.7]	24.2 [19.3; 29.2]	-12.14 [-28.64; 5.06]	-14.34 [-30.62; 2.15]
10 - 11	31.7 [28.2; 35.7]	35.8 [32.0; 40.1]	24.2 [19.2; 29.1]	23.7 [18.5; 28.8]	-23.67 [-39.44; -7.52]	-29.28 [-43.27; -14.36]
11 - 12	29.2 [25.9; 33.0]	35.1 [31.3; 39.3]	24.1 [18.9; 29.2]	22.8 [17.4; 28.1]	-12.08 [-31.05; 6.64]	-33.07 [-49.39; -17.44]
12 - 13	32.1 [28.6; 36.0]	29.5 [26.1; 33.3]	23.7 [18.3; 29.0]	21.5 [16.0; 27.0]	-16.43 [-33.07; -0.24]	-25.68 [-43.93; -6.99]
13 - 14	30.4 [27.0; 34.1]	20.3 [17.5; 23.5]	23.1 [17.5; 28.6]	20.1 [14.4; 25.7]	-17.53 [-36.19; 0.78]	-2.02 [-29.52; 24.65]
14 - 15	28.8 [25.5; 32.5]	16.4 [13.9; 19.3]	22.2 [16.6; 27.9]	18.4 [12.6; 24.1]	-37.83 [-56.31; -19.41]	13.64 [-21.57; 48.81]
15 - 16	21.8 [19.0; 25.0]	13.9 [11.6; 16.6]	21.3 [15.5; 27.1]	16.6 [10.7; 22.4]	2.96 [-23.74; 28.97]	-12.52 [-54.94; 30.19]
16 - 17	17.1 [14.7; 20.0]	13.1 [10.9; 15.7]	20.2 [14.2; 26.2]	14.7 [8.68; 20.6]	13.85 [-20.99; 49.64]	-18.22 [-62.38; 27.72]
17 - 18	12.4 [10.3; 14.9]	12.5 [10.4; 15.1]	19.0 [13.0; 25.2]	12.8 [6.66; 18.8]	48.04 [-0.50; 97.44]	-3.35 [-50.35; 43.71]
18 - 19	9.4 [7.58; 11.6]	6.67 [5.15; 8.62]	17.8 [11.6; 24.1]	10.8 [4.62; 17.0]	71.08 [5.51; 135.70]	73.32 [-14.84; 163.86]
19 -20	3.29 [2.27; 4.77]	2.30 [1.47; 3.61]	16.6 [10.3; 23.0]	8.87 [2.57; 15.1]	319.04 [131.56; 522.32]	217.11 [-46.18; 505.81]

S2 Table. Observed and estimated average incidence rates of type 2 diabetes

Age	Average observed incidence rate (per 100.000 person-years)		Average estimated incidence rate (per 100.000 person-years)		Relative error (in %)	
	Male	Female	Male	Female	Male	Female
0 - 1	0.00 [0.00; 0.00]	0.00 [0.00; 0.00]	0.00 [0.00; 0.00]	0.00 [0.00; 0.00]	NA	NA
1 - 2	0.20 [0.05; 0.79]	0.00 [0.00; 0.00]	0.00 [0.00; 0.00]	0.00 [0.00; 0.00]	NA	NA
2 - 3	0.00 [0.00; 0.00]	0.00 [0.00; 0.00]	0.00 [0.00; 0.00]	0.05 [0.00; 0.31]	NA	NA
3 - 4	0.10 [0.01; 0.71]	0.00 [0.00; 0.00]	0.14 [0.00; 0.37]	0.80 [0.42; 1.23]	NA	NA
4 - 5	0.00 [0.00; 0.00]	0.32 [0.10; 0.98]	0.56 [0.24; 0.91]	1.59 [1.02; 2.19]	NA	-64.11 [-1041.18; 654.11]
5 - 6	0.10 [0.02; 0.71]	0.65 [0.29; 1.43]	1.03 [0.57; 1.50]	2.43 [1.67; 3.20]	-72.29 [-1283.21; 1209.03]	-15.51 [-672.26; 668.63]
6 - 7	0.30 [0.10; 0.94]	0.58 [0.25; 1.34]	1.55 [0.95; 2.15]	3.31 [2.36; 4.28]	-49.13 [-1172.20; 894.05]	165.75 [-2760.45; 2730.97]
7 - 8	0.10 [0.01; 0.71]	0.53 [0.22; 1.26]	2.13 [1.38; 2.88]	4.24 [3.10; 5.39]	-65.82 [-1230.74; 1139.92]	349.83 [-5010.38; 6005.12]
8 - 9	1.02 [0.55; 1.89]	1.70 [1.05; 2.77]	2.77 [1.86; 3.67]	5.20 [3.86; 6.54]	69.17 [-26.13; 340.70]	102.79 [20.34; 225.85]
9 - 10	0.80 [0.40; 1.60]	3.34 [2.36; 4.72]	3.46 [2.40; 4.52]	6.16 [4.62; 7.68]	210.63 [28.78; 1522.65]	75.60 [23.94; 129.79]
10 - 11	2.98 [2.10; 4.24]	7.82 [6.27; 9.77]	4.19 [3.00; 5.42]	7.08 [5.35; 8.78]	50.60 [2.63; 102.08]	7.79 [-17.50; 33.59]
11 - 12	3.23 [2.30; 4.52]	9.90 [8.13; 12.1]	4.94 [3.55; 6.33]	7.92 [6.02; 9.81]	24.16 [-18.69; 70.24]	-25.35 [-43.57; -7.18]
12 - 13	6.56 [5.19; 8.29]	12.6 [10.6; 15.0]	5.70 [4.13; 7.25]	8.66 [6.58; 10.7]	-18.30 [-40.96; 4.57]	-1.39 [-20.10; 17.44]
13 - 14	10.1 [8.38; 12.2]	13.5 [11.4; 15.9]	6.42 [4.69; 8.15]	9.26 [7.02; 11.5]	-21.55 [-39.49; -4.03]	-17.35 [-33.76; -0.84]
14 - 15	10.9 [9.14; 13.1]	14.4 [12.2; 16.9]	7.10 [5.20; 9.00]	9.72 [7.31; 12.1]	-21.34 [-38.62; -4.04]	-15.19 [-31.57; -13.40]
15 - 16	9.90 [8.20; 12.0]	14.4 [12.2; 16.9]	7.71 [5.64; 9.78]	10.0 [7.46; 12.6]	7.52 [-15.22; 30.71]	-13.40 [-31.61; 5.26]
16 - 17	10.8 [9.01; 12.9]	15.4 [13.2; 18.0]	8.26 [6.02; 10.5]	10.2 [7.50; 12.9]	-4.76 [-26.02; 17.06]	-25.38 [-42.61; -7.53]
17 - 18	11.0 [9.17; 13.1]	13.1 [11.0; 15.5]	8.74 [6.34; 11.1]	10.3 [7.44; 13.2]	-26.83 [-49.58; -3.56]	-34.06 [-56.92; -11.11]
18 - 19	9.11 [7.47; 11.1]	9.43 [7.71; 11.5]	9.19 [6.63; 11.7]	10.4 [7.34; 13.4]	-7.40 [-34.55; 19.75]	-9.67 [-40.63; 22.45]
19 -20	3.99 [2.92; 5.45]	5.23 [3.96; 6.91]	9.61 [6.90; 12.3]	10.4 [7.21; 13.6]	78.02 [13.17; 147.91]	42.16 [-16.32; 101.94]
